# Metabolic changes upon flower bud break in Japanese apricot are enhanced by exogenous GA_4_

**DOI:** 10.1038/hortres.2015.46

**Published:** 2015-09-30

**Authors:** Weibing Zhuang, Zhihong Gao, Luhua Wen, Ximei Huo, Binhua Cai, Zhen Zhang

**Affiliations:** 1 College of Horticulture, Nanjing Agricultural University, Nanjing 210095, China

## Abstract

Gibberellin (GA_4_) has a significant effect on promoting dormancy release in flower buds of Japanese apricot (*Prunus mume* Sieb. et Zucc). The transcriptomic and proteomic changes that occur after GA_4_ treatment have been reported previously; however, the metabolic changes brought about by GA_4_ remain unknown. The present study was undertaken to assess changes in metabolites in response to GA_4_ treatment, as determined using gas chromatography–mass spectrometry and principal component analysis. Fifty-five metabolites that exhibited more than two-fold differences in abundance (*P* < 0.05) between samples collected over time after a given treatment or between samples exposed to different treatments were studied further. These metabolites were categorized into six main groups: amino acids and their isoforms (10), amino acid derivatives (7), sugars and polyols (14), organic acids (12), fatty acids (4), and others (8). All of these groups are involved in various metabolic pathways, in particular galactose metabolism, glyoxylate and dicarboxylate metabolism, and starch and sucrose metabolism. These results suggested that energy metabolism is important at the metabolic level in dormancy release following GA_4_ treatment. We also found that more than 10-fold differences in abundance were observed for many metabolites, including sucrose, proline, linoleic acid, and linolenic acid, which might play important roles during the dormancy process. The current research extends our understanding of the mechanisms involved in budburst and dormancy release in response to GA_4_ and provides a theoretical basis for applying GA_4_ to release dormancy.

## Introduction

Bud dormancy is a complex process in woody perennials that enables plants to survive long periods in adverse conditions, including extremes of cold, drought, and heat.^1^ Dormancy, which coincides with winter, is controlled by the bud itself and is released by exposure to chilling. Therefore, to resume growth, the tree buds must receive a specific amount of chilling; the required amount is genetically controlled and varies among genotypes.^2^ Warm winters in many regions often limit the growth of plants and cause a reduction in the productivity of temperate fruit crops. To overcome this problem, artificial dormancy-breaking agents are applied to regulate dormancy release and to compensate for insufficient chilling.

Several chemicals such as hydrogen cyanamide, mineral oil, and potassium nitrate can be used to induce bud break of deciduous fruit trees in areas that lack sufficient chilling.^3–5^ However, the available compounds are costly and may have a risk of bud damage owing to their phytotoxicity.^6–8^ Recently, Rinne *et al*. reported that GA_4_ can induce true budburst in *Populus*, and we also observed that GA_4_ treatment can replace the requirement for chilling and accelerate bud break in Japanese apricot.^9,10^ In addition, owing to its low toxicity toward the human body, GA_4_ holds the promise of being broad applicable to breaking dormancy in deciduous fruit trees. Therefore, studying the mechanism of GA_4_-induced dormancy release has many potential applications.

Many studies have focused on the biochemical changes in dormant tissues in deciduous fruit trees. Tan *et al*. studied the respiratory response in dormant nectarine vegetative buds under high-temperature stress conditions, and Seif El-Yazal and Rady observed that increased biogenic amine and proline content contributed to bud dormancy release in ‘Ain Shemer’ apple trees.^11–13^ Mohamed *et al.* studied the changes in carbohydrates of superior seedless grapevine cuttings during dormancy release after hydrogen cyanamide treatment, and Ito *et al*. determined the carbohydrate metabolism levels during the dormancy transition in Japanese pear.^14,15^ Accumulating evidence has indicated that signaling mechanisms are associated with metabolism play important roles in regulating the transition between dormancy and growth.^16^ Photosynthesis, which acts as a low-temperature sensor, can serve as a key integrator of low temperature and signaling outputs such as antioxidants, sugars, and reactive oxygen species (ROS) as well as redox state.^17^ The status of energy flow from a source to a metabolic sink is sensed by the redox state of electron transport chain components, which contribute to the reduction of redox transmitters, ROS, and antioxidants, and these changes can alter gene expression at the transcriptional and post-transcriptional levels.^17–19^ Antioxidant systems and redox systems, such as ROS, have been reported to play roles in regulating bud dormancy release.^20–23^ Carbohydrate metabolism also plays an important role in controlling the dormancy process.^24^ For example, an increase in sucrose phosphate synthase activity, which leads to sucrose synthesis, is often observed under low-temperature conditions.^17,25^ However, the metabolic changes in dormant Japanese apricot flower buds treated with GA_4_, as determined using a metabolomics approach, have not been reported.

Metabolomics approaches, which are the latest addition to the functional genomics toolbox, can be used to detect a wide range of metabolites simultaneously. Therefore, these approaches can provide a comprehensive quantitative list of metabolites that can be mapped to specific pathways. Metabolomics can also act as an amplifier of transcriptomic and proteomic changes.^16^

Japanese apricot (*Prunus mume* Sieb. et Zucc), which originated in China, has a long cultivation history in Asia, approximately 3000 years.^26^ Owing to its broad chilling requirement range, many researchers use this species to study the mechanism of dormancy.^10,27–33^ The recently available genome sequence of *P. mume* can also contribute to our understanding of dormancy and provide additional data to explore the mechanism of dormancy.^34^ Increasing numbers of scientists have begun to use metabolomics approaches to study molecular mechanisms in fruit trees.^35–38^ However, to date, there is no report in which a metabolomic approach has been used to study the mechanisms of dormancy release in Japanese apricot.

We previously investigated the active role of GA_4_ in Japanese apricot flower bud dormancy release at the proteomic and transcriptomic levels.^10^ As previously reported by Zhuang, the percentages of flower buds that showed burst were as follows: W0 (water treatment at 0 days, 0% ± 0.0%), G5 (GA_4_ treatment at 5 days, 15.0% ± 1.5%), W5 (water treatment at 5 days, 5.0% ± 0.6%), G10 (GA_4_ treatment at 10 days, 60.0% ± 2.5%), and W10 (water treatment at 10 days, 20.0% ± 1.0%). These results indicate that GA_4_ treatment plays an important role in dormancy release in Japanese apricot.^10^ In the present study, we used gas chromatography–mass spectrometry (GC–MS) to evaluate the metabolic changes in the Japanese apricot flower bud after GA_4_ treatment. The primary objective of this work was to obtain a comprehensive and dynamic metabolic profile of Japanese apricot treated with GA_4_. The second objective was to identify metabolites which changed a lot in abundance in response to GA_4_ treatment during the dormancy process. The third objective was to explore the metabolic pathways that are important in dormancy release upon GA_4_ treatment.

## Materials and methods

### Plant materials and treatment

The Japanese apricot cultivar ‘Bungo’ is a late flowering cultivar that was grown in the National Field Genebank for Japanese apricot in Nanjing, Jiangsu Province, China. We conducted GA_4_ and water treatments in accordance with our previous study.^10^ Briefly, 1-year-old branches with a length of approximately 40 cm, and a diameter of approximately 5 mm (containing approximately 25 buds) that were located in the middle portion of trees were cut from ‘Bungo’ trees on 30 December 2011. GA_4_ (100 μM) was supplied to the bud via the stem vasculature and was not directly applied to the bud, in accordance with the method described by Rinne.^9^ The branch bases were placed in water containing GA_4_ or in water without GA_4_ as a control and incubated in a growth chamber. The branches were maintained under white fluorescent light (55 μmol m^−2^ s^−1^), with a photoperiod of 16 h light at 25 ± 1 °C and 8 h dark at 18 ± 1 °C, and a constant relative humidity of 70%. After 2 days, the solution was changed, and the base of each branch was cut away. The branches were retained in the growth chamber for 10 days, after which the flower bud break percentage was counted. The branch cuttings were defined as breaking dormancy when 50% of the flower buds on the branch cuttings were in the green tip stage.^39^ More than 120 flower buds were assessed for each treatment. Flower buds were collected from the middle portions of the branches at 0, 5, and 10 days after treatment and were immediately frozen in liquid nitrogen and stored at –70 °C for further use. G0/W0 represents GA_4_ or water treatment at 0 day; W5 and W10 represent water treatment at 5 and 10 days, respectively; and G5 and G10 represent GA_4_ treatment at 5 and 10 days, respectively. We collected six samples for each stage (W0, W5, W10, G5, and G10), and each sample represented a pool of flower buds from three trees (nine flower buds).

### Chemicals

Methanol and chloroform (high-performance liquid chromatography grade) were purchased from Fisher Scientific (Hampton, NH, USA). GC grade pyridine, methoxyamine pyridine, and internal standards (the reference ribitol) were purchased from Sigma-Aldrich (St. Louis, MO, USA). N,O-Bis(trimethylsilyl)trifluoroacetamide (BSTFA) reagent (containing 1% trimethylchlorosilane (TMCS)) was purchased from Regis Technologies Inc., (Morton Grove, IL, USA). Deionized water was purified in-house using a Millipore Milli-Q system (Millipore Corporation, Billerica, MA, USA).

### Metabolite extraction

Metabolites from Japanese apricot flower buds (50 mg fresh weight) were extracted as reported by Lisec *et al*.^40^ with some modifications as follows. Flower buds stored at –70 °C were grounded in a mortar in liquid nitrogen and transferred to 2 mL centrifuge tubes. In total, 1.4 mL 100% methanol (pre-cooled to –20 °C) was added to the samples, and the samples were vortexed for 10 s. Then, 60 μL ribitol (0.2 mg mL^–1^ stock in deionized water) was added to each sample as an internal quantitative standard, and the samples were vortexed for 10 s. The tubes were exposed to ultrasound at 70 °C for 30 min and then centrifuged for 10 min at 11 000*g*. The supernatant was transferred to 5 mL glass centrifuge tubes. After the addition of 750 μL chloroform (pre-cooled to –20 °C) and 1.5 mL deionized water (4 °C), the tubes were vortexed for 30 s and then centrifuged for 15 min at 2200*g*. Subsequently, 400 μL supernatant was transferred into a new Eppendorf tube, and the samples were dried under a moderate nitrogen flow.

### Derivatization and GC–MS analysis

For GC–MS analysis, 80 μL 15 mg mL^–1^ methoxyamine pyridine solution was added to the samples, which were vortexed for 30 s and allowed to react for 90 min at 37 °C. Finally, 80 μL BSTFA reagent (containing 1% TMCS) was added to the mixture, which was incubated for 60 min at 70 °C. Following these reactions, samples were analyzed for metabolite content using an Agilent 7890 gas chromatograph system coupled with a Pegasus 4D time-of-flight mass spectrometer. The system utilized a DB-5 ms capillary column coated with 5% diphenyl cross-linked with 95% dimethylpolysiloxane (30 m × 250 μm inner diameter, 0.25-μm film thickness; J&W Scientific, Folsom, CA, USA). A 1 μL aliquot of the analyte was injected in splitless mode. Helium was used as the carrier gas, the front inlet purge flow was 3 mL min^–1^, and the gas flow rate through the column was 1 mL min^–1^. The initial temperature was retained at 60 °C for 0.5 min, then raised to 100 °C at a rate of 16 °C min^−1^, and finally to 300 °C at a rate of 5 °C min^–1^ for 5 min. The injection, transfer line, and ion source temperatures were 280 °C, 260 °C, and 230 °C, respectively. The energy was −70 eV in electron impact mode. The MS data were acquired in full-scan mode with an m/z range of 50–500 at a rate of 150 spectra per second after a solvent delay of 550 s.

### Data acquisition, deconvolution, and peak identification

User-defined libraries were generated using the automated mass-spectral deconvolution and identification system (AMDIS; National Institute of Standards) to deconvolute GC-MS results and identify distinct chromatographic components. Retention indices (RIs) were generated for each sequence by comparing the retention times of sample components with the retention times of C10–C40 alkanes evaluated under the same conditions as the samples. Individual libraries made from samples exposed to each treatment were compared, and redundant components were eliminated. From these libraries, mass spectral tags were cataloged using the RIs coupled with key mass spectral features, and calibration tables were generated using ChemStation (G1701DA rev. D; Agilent Technologies, Palo Alto, CA, USA). The Qedit macro was used to evaluate each compound and to provide peak areas for the components. Mass spectral comparison with spectral catalogs in NIST05 (National Institute of Standards) and mass spectral interpretation aided in the tentative identification of many of the components. Compound identifications are based on comparisons of sample compound spectra and RIs with those of authentic standards. Chroma TOF 4.3X software (LECO, St. Joseph, MI, USA) was used for auto acquisition of GC total ion chromatograms (TICs) and fragmentation patterns. Each compound had a unique fragmentation pattern composed of a series of split molecular ions, the mass–charge ratios and the abundance of which was compared with a standard mass chromatogram in the LECO-Fiehn Rtx5 mass spectra library by using the Chroma TOF 4.3X software. For each peak, the software generated a list of similarities by comparison with the peaks of every substance within the LECO-Fiehn Rtx5 library. Additionally, peaks with a similarity of more than 700 were selected for further research.^41^ The metabolite abundance was expressed according to the relative area values of the peaks, and the ratio of the peak area of each compound to the corresponding internal standard (ribitol) was calculated as the response.

### Statistical analyses

The target ion response data of the peak areas for mass-extracted ion chromatograms were corrected by comparing ribitol in each sample with that in an external standard and the sample fresh weight. These values were transformed by mean centering and log_10_-transformation before principal component analysis (PCA) using the SAS 9.1 software package (SAS Institute Inc., Cary, NC, USA). By modeling the data using PCA, the variance in a multivariate data set containing many instrument-derived components is reduced to a few orthogonal variables, called principal components, that each accounts for a portion of the variance in the data set. By plotting the treatment scores or component loading values derived from PCA in the space defined by the largest principal components, associations between treatments and metabolic components can be revealed. For each cultivar, the data were analyzed together, and treatment differences were evaluated by plotting principal component scores against each other. Only metabolites that showed a more than two-fold or less than 0.5-fold change and that had statistically significant, reproducible changes in six analytical replicates, as determined by two-way analysis of variance (ANOVA) (*P* < 0.05), were considered for subsequent analysis. The analysis was performed using SAS version 9.1 (SAS Institute Inc.). Metabolic pathways were constructed according to the KEGG metabolic database.

### Total RNA extraction

Total RNA was extracted from flower buds (100 mg) using the cetyltrimethyl ammonium bromide method.^10^ Genomic DNA contamination was removed with RNase-free DNase I (TaKaRa, Japan) according to the manufacturer’s instructions. The RNA was stored at −70 °C until use.

### Real-time PCR validation

The expression of candidate genes was determined using real-time PCR (RT-PCR). Total RNA (1 μg) samples were reverse-transcribed for first-strand cDNA synthesis using a ReverTra Ace qPCR RT Kit according to the manufacturer’s instructions. According to the sequence of the target gene, which was downloaded from http://www.biomedsearch.com/nih/genome-Prunus-mume/23271652.html, we used Primer Premier 5.0 software to design the primers (Supplementary Table S1). RT-PCR was performed with a 20 μL reaction volume on an Applied Biosystems 7300 Real-Time PCR System. Each reaction contained 0.3 μL (10 pM) each primer, 8.4 μL sterile double-distilled water, 10 μL SYBR Premix Ex Taq, and 1 μL 10-fold diluted cDNA. The RT-PCR conditions were as follows: 95 °C for 3 min followed by 40 cycles of 95 °C for 25 s, 62 °C for 25 s, and 72 °C for 40 s. Gene expression was analyzed using the 2^−ΔCT^ method, which represents the difference in the cycle threshold (CT) between the target gene products and control RP II products.^42^ Data analyses were performed using SPSS version 17.0 statistical software. Triplicate samples were used for RT-PCR.

## Results

### PCA of metabolic profile changes

**Figure 1:**
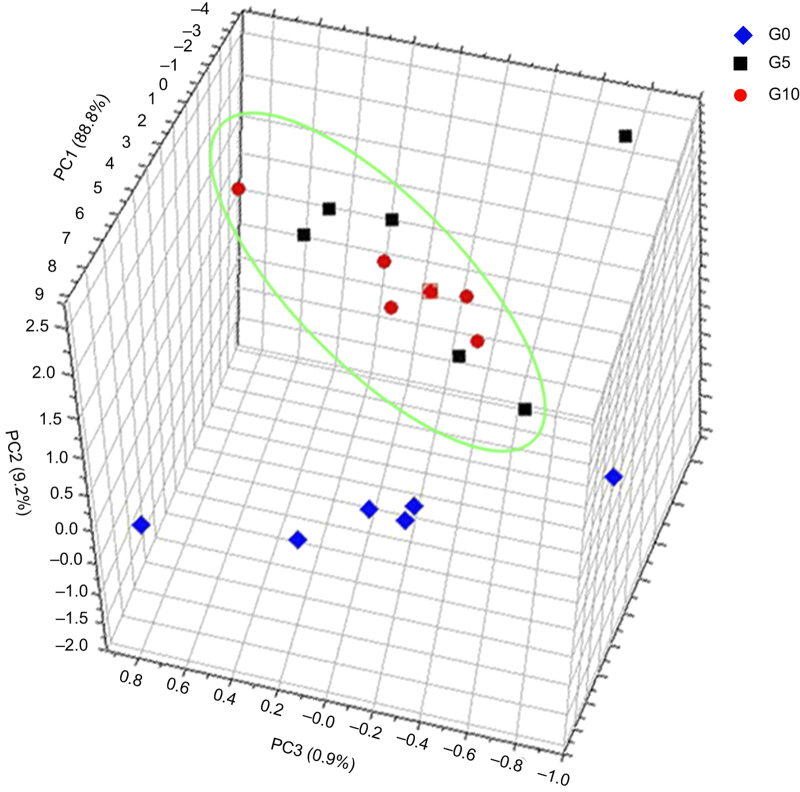
Three-dimensional PCA score plot for GA_4_ treatment, showing clear differences between 0 and 5 days of GA_4_ treatment and no clear difference between 5 and 10 days of GA_4_ treatment. This result indicates variation in metabolites in response to GA_4_ treatment. G0, G5, and G10 denote GA_4_ treatment after 0, 5, and 10 days, respectively.

**Figure 2:**
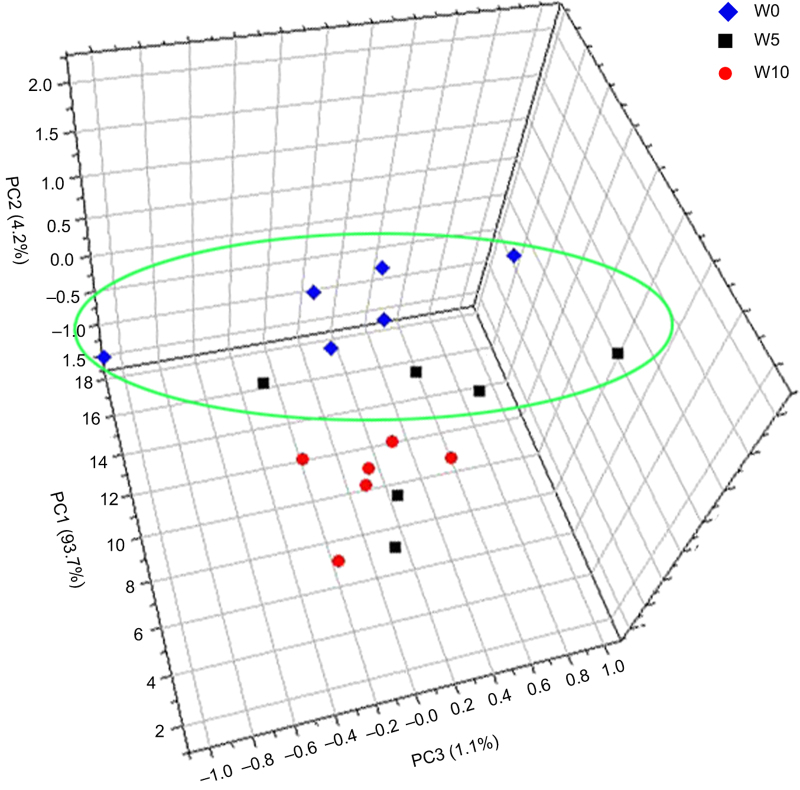
Three-dimensional PCA score plot for water treatment, showing no differences between 0 and 5 days of water treatment and clear differences between 0 and 10 days of water treatment. This result indicates variation in metabolites in response to water treatment. W0, W5, and W10 denote water treatment after 0, 5, and 10 days, respectively.

To verify the reliability of metabolomic analysis, we obtained the GC–MS TIC for 42 Japanese apricot flower bud samples. An obvious chromatographic difference was observed between sample groups, and the retention times were reproducible and stable, indicating the reliability of metabolomic analysis (Supplementary Figure S1). We also generated three-dimensional PCA plots for metabolic profile changes, to systematically assess the metabolic responses due to GA_4_ treatment. Figure 1 shows a clear separation between G0 and G5, and no significant separation between G5 and G10, suggesting that 5 days of GA_4_ application led to many changes in metabolites (compared with 0 days of GA_4_ application) and that only a few metabolites changed in abundance between 5 and 10 days of GA_4_ application. We also observed a clear separation between W0 and W10, and no clear separation between W0 and W5, indicating that clear metabolic differences existed after 10 days of water treatment and that few metabolic differences existed between 5 and 0 days of water treatment (Figure 2).

### Metabolic alterations in response to GA_4_ treatment in Japanese apricot

**Figure 3:**
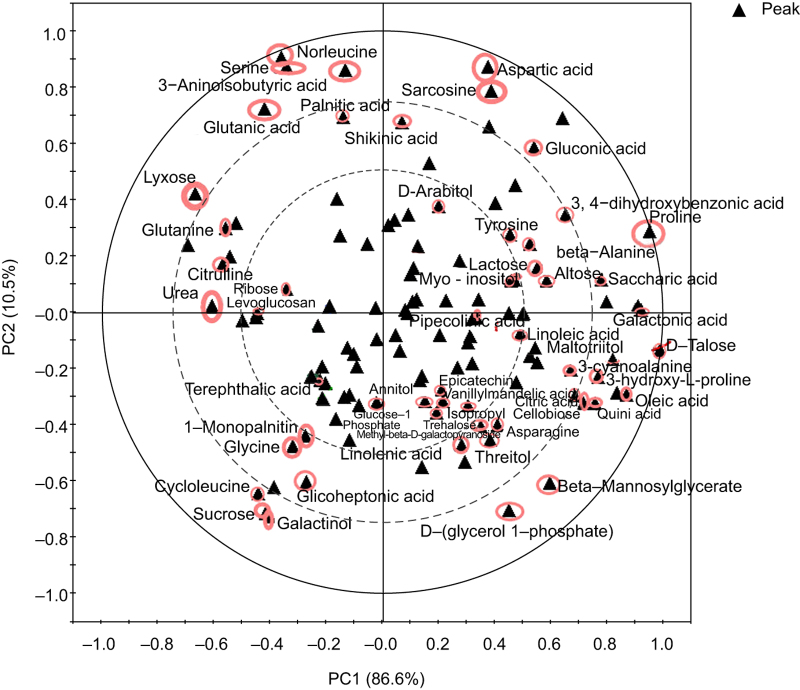
PCA of the metabolic profiles of flower buds after GA_4_ treatment or water treatment. The score and loading plots for the first and second principal components are shown, and the loading plots show the contribution of the different metabolites to the sample projection. The detected metabolites are marked with solid triangles, and the 55 metabolites showing a more than two-fold increase/decrease after treatment are marked with red circles.

The abundances of some metabolites were altered after GA_4_ treatment. Sixteen metabolites had a higher abundance after 5 days of GA_4_ treatment, and 21 had a lower abundance, and after 10 days of GA_4_ treatment, three metabolites had a higher abundance than at 5 days and 15 had a lower abundances (Supplementary Figure S2). These findings indicated that the abundances of more metabolites were altered (by more than two-fold) after 5 days of GA_4_ treatment than after 10 days of GA_4_ treatment and presumably played roles in dormancy release at 5 days of GA_4_ treatment. After 5 days of water treatment, the abundances of 12 metabolites were higher and 21 were lower, and after 10 days of water treatment, 20 were higher and 14 were lower (Supplementary Figure S2), indicating that a similar number of metabolites were altered in abundance during 5 and 10 days’ water treatment of Japanese apricot. The metabolites detected to have more than two-fold increases/decreases in abundance following GA_4_ or water treatment are shown in Figure 3; these metabolites detected after GA_4_ treatment may play important roles in dormancy release.

Dormancy was released in branch cuttings after 10 days of GA_4_ treatment, which was earlier than in branches treated with water. The metabolites with more than two-fold abundance changes following GA_4_ treatment were classified into six groups: amino acids and their isoforms (8), amino acid derivatives (6), sugars and polyols (12), organic acids (8), fatty acids (3), and others (7) (Table 1). These metabolites were involved in carbohydrate metabolism (e.g., citric acid and lactose), lipid metabolism (e.g., oleic acid and linolenic acid), and amino acid metabolism (e.g., tyrosine and glycine), all of which are processes that might provide energy for dormancy release and sustain subsequent growth.

**Figure 4:**
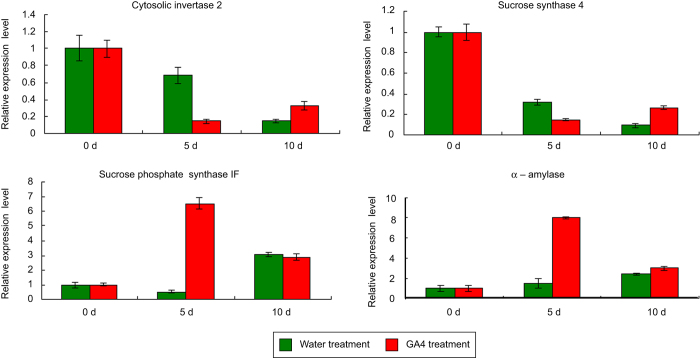
The relative expression levels of four genes, as determined by quantitative RT-PCR at 0, 5, and 10 days of GA_4_ treatment or water treatment, in flower buds of Japanese apricot. The expression levels were normalized to RNA polymerase subunit expression. Bars indicate the mean ± SD, *n* = 3.

In our study, the abundances of some of the 55 metabolites that showed a more than two-fold increase/decrease after treatment changed more than 10-fold after GA_4_ treatment; these metabolites included sucrose, proline, linoleic acid, and linolenic acid, which might play roles in the dormancy process. After 5 days of GA_4_ treatment, the sucrose content had increased 13.86-fold over the level at 0 days of water treatment, whereas after 5 days of water treatment, the sucrose content showed only a 1.77-fold increase compared to the sucrose level at 0 days of water treatment. After 10 days of GA_4_ treatment, the sucrose levels had decreased 7.50-fold compared with 5 days of GA_4_ treatment. In contrast, after 10 days of water treatment, the sucrose content had increased 17.01-fold compared with 5 days of water treatment (Table 1 and Supplementary Table S2). We also studied the trends in the expression of three genes associated with sucrose metabolism (*INVERTASE*, *SUCROSE-6-PHOSPHATE SYNTHASE* (*SPS*), and *SUCROSE SYNTHASE* (*SS*)). *SPS*, a gene associated with sucrose synthesis, was significantly up-regulated after 5 days of GA_4_ treatment, while this gene was slightly down-regulated after 5 days of water treatment. After 10 daysof GA_4_ treatment, SPS was down-regulated compared with after 5 days of GA_4_ treatment, while after 10 days of water treatment, SPS was up-regulated compared with after 5 days of water treatment. *INVERTASE* and *SS*, two genes responsible for sucrose breakdown, showed similar trends during the water treatment and GA_4_ treatment. *INVERTASE* and *SS* were significantly down-regulated after 5 days of GA_4_ treatment, while these genes were only slightly down-regulated after 5 days of water treatment. After 10 days of GA_4_ treatment, *SPS* was up-regulated compared with after 5 days of GA_4_ treatment, while after 10 days of water treatment, *SPS* continued to down-regulated compared with that after 5 days of water treatment. The trends in the expression of these three genes were consistent with the changes in sucrose content (Figure 4). The proline concentration showed a change similar to that of sucrose after GA_4_ or water treatment, and the proline content was higher after GA_4_ treatment than after water treatment (Table 1 and Supplementary Table S2).

After 5 and 10 days of GA_4_ treatment, the abundance of linoleic acid decreased 2.24-fold and 133.09-fold compared with 0 and 5 days, respectively, while after 5 and 10 days of water treatment, the abundance of linoleic acid decreased 4.06-fold and 1.16-fold, respectively (Table 1 and Supplementary Table S2). After 5 and 10 days of GA_4_ treatment, the abundance of linolenic acid decreased 22.69-fold and increased 23.26-fold compared with 0 and 5 days of GA_4_ treatment, respectively, while after 5 and 10 days of water treatment, the abundance of linolenic acid decreased 2.67-fold and increased 2.61-fold, respectively (Table 1 and Supplementary Table S2). The changes in metabolite abundance after GA_4_ treatment were similar to those in response to water treatment. However, GA_4_ treatment induced faster changes than did water treatment. In our study, most metabolites associated with fatty acids increased/decreased their abundance more than 10-fold after GA_4_ treatment, which suggested that metabolites associated with fatty acids might be involved in dormancy release.

### Metabolic pathways associated with dormancy release after GA_4_ treatment

**Figure 5:**
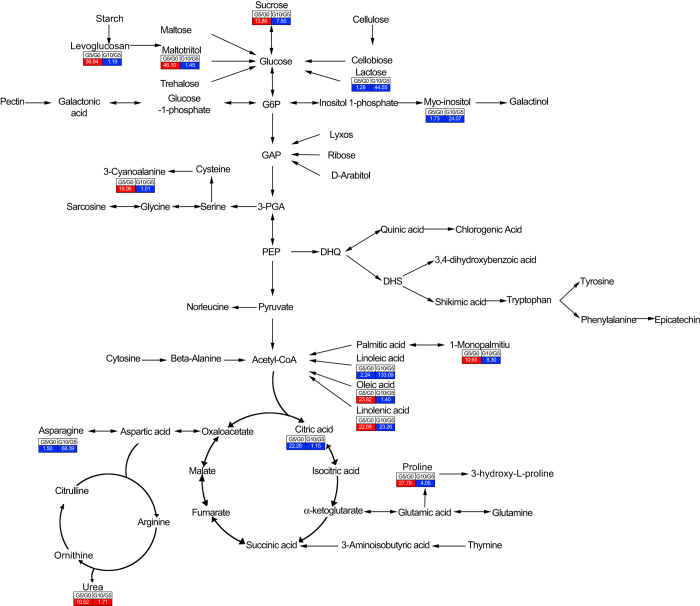
Changes in metabolites of the metabolic pathways in flower buds after GA_4_ (**a**) and water treatment (**b**) in Japanese apricot. Schematic summary of the tricarboxylic acid (TCA) cycle and its convergent and divergent pathways showing that the TCA cycle is embedded in a complex metabolic network and that energy metabolism might function in flower bud dormancy release. The mean values of six independent determinations for each treatment are expressed as relative concentration. Only those metabolites that showed a more than 10-fold or less than 0.1-fold change are shown. The number in the box shows the fold changes in metabolites with a changed abundance after treatment. Red indicates increased metabolite content after treatment, and blue indicates decreased metabolite content after treatment. W0, W5, and W10 denote water treatment after 0, 5, and 10 days, respectively. G0, G5, and G10 denote GA_4_ treatment after 0, 5, and 10 days, respectively.

Figure 5 represents most of the metabolites identified to have more than 10-fold abundance changes across the entire metabolic cycle. These metabolites participate in various metabolic pathways, such as galactose metabolism, starch and sucrose metabolism, glyoxylate and dicarboxylate metabolism, and pentose and glucuronate interconversions. We also observed that many more of these metabolites were involved in galactose, starch, sucrose, glyoxylate, and dicarboxylate metabolism than in other metabolic pathways (Supplementary Figures S3–S5), which indicated that energy metabolism might play an important role in GA_4_-induced dormancy release at the metabolic level.

## Discussion

Dormancy is a complex phenomenon that allows trees to adapt better to unfavorable conditions during winter. Although plants suspend their visible growth in winter, developmental changes still occur, and buds are biochemically and physiologically active.^43^ Numerous physiological changes, such as changes in respiration rate, carbohydrate metabolism, growth regulator levels, water content, and the activities of other compounds thought to be involved in dormancy release, have been studied to analyze the control of dormancy.^1,44^ GA_3_ treatment has been shown to be a good method for breaking bud dormancy in some trees. Recently, another biologically active gibberellin, GA_4_, was also found to play important roles during dormancy release in *Populus* and Japanese apricot. GA_4_ was found to be more effective than was GA_3_ at stimulating seed germination in *Arabidopsis thaliana* under dark conditions.^45^ Zhang et al. investigated the temporal variations in endogenous GA_3_ and GA_4_ in the leaves of birch (*Betula platyphylla*) and found that GA_3_ and GA_4_ had different effects on promoting birch growth and flowering.^46^ Although both GA_3_ and GA_4_ belong to the biologically active gibberellin class, they promote different mechanisms of bud dormancy release. GA_3_ treatment can up-regulate all LB-associated GH17s, whereas GA_4_ treatment can up-regulate most GH17s with a GPI anchor and/or callose binding motif.^9^ Therefore, we believe that the dormancy release effect of GA_4_ is specific and cannot be reproduced by other active GAs. Thus far, few studies have examined dormancy release in response to other biologically active gibberellins such as GA_1_ and GA_9_.

Many reports have shown that bud dormancy release is accompanied by a short period of high proline accumulation.^14,47,48^ Free proline accumulation is a common response to various stresses in plants; this response can maintain the osmotic balance between the cytosol and the vacuole. Owing to proline’s ability to remove ROS, which are generated by stress in plants, proline can also serve as an osmoprotectant of subcellular structures. Kishor *et al.* observed extensive proline accumulation under stress conditions and a rapid decrease in proline accumulation after the stress was relieved.^49^ However, Ruttink *et al*. performed metabolomics analyses during poplar dormancy initiation and found that the proline levels do not significantly change, which indicated that proline might not play a role in poplar during dormancy initiation.^50^ In contrast, in our study, GA_4_ application led to a significant but transient increase in proline content, which peaked at 5 days of GA_4_ treatment before decreasing (Table 1). The idea that oxidative stress causes proline accumulation further supports the assumption that GA_4_ induces transient oxidative stress, which is consistent with our previous report that proteins and genes associated with oxidation–reduction also play an important role after GA_4_ treatment of Japanese apricot.

The increase in proline biosynthesis, in combination with the high peroxidase activity after GA_4_ treatment that precedes bud break (as shown by our previous results), might lead to accumulation of NADP^+^ and thus an increase in the NADP^+^/NADPH ratio. This increase probably activates the oxidative pentose phosphate pathway (PPP), which is a major source of NADPH in non-photosynthetic tissues.^51^ Oxidative stress lead to the activation of the PPP, which generates more NADPH for proline biosynthesis and activates other pathways, including antioxidant responses in peroxidases.^52^ Furthermore, proline-linked PPP activity has been speculated to provide the phosphate sugars and NADPH that are used for antioxidant pathway activity and phenolic synthesis, e.g., via the ascorbate–glutathione cycle (AGC).^52^ Therefore, the transient induction of proline synthesis observed here, in addition to peroxidase and ascorbate peroxidase activities (reported by our previous study), might be associated with the induction of the AGC and PPP during dormancy release.^10^ Based on these findings, we can hypothesize that the stimulation of proline-linked PPP activity might function in GA_4_-induced dormancy release. However, further studies are needed to confirm this possibility.

Carbohydrates are the primary source of energy for the metabolic changes that occur in plants during the dormancy release period. Carbohydrate availability is considered to be of major relevance to the control of bud growth and dormancy.^53^ In our study, the level of sucrose increa4sed 13.86-fold after 5 days of GA_4_ treatment compared with 0 days but only 1.77-fold after 5 days of water treatment, indicating that GA_4_ might lead to a rapid increase in sucrose and therefore release dormancy, similar to results obtained by Mason *et al.*^54^ The sucrose content reached its maximum value earlier in response to GA_4_ treatment than to water treatment, which may be one of the reasons that GA_4_ treatment can break bud dormancy earlier. In many cases, sucrose serves as a source of energy and carbon during the developmental process. However, sucrose can also serve as a potent signaling molecule that regulates physiology and development. Hanson observed that sucrose, as a signaling molecule, regulates the transcription factor bZIP11, which affects amino acid metabolism by regulating the expression of *PROLINE DEHYDROGENASE 2* and *ASPARAGINE SYNTHETASE 1*.^55^ Radchuk *et al.* reported that sucrose affects carbon fluxes at the transcriptional and post-transcriptional levels.^56^ Ruttink *et al*. also found that the low sugar levels downstream of an SD response might generate endogenous signals (such as ethylene) that subsequently contribute to progression to the next phase of bud development.^50^ Ruan reported the signaling role of sucrose metabolism in development.^57^ Mason *et al*. reported that sugars are rapidly redistributed over large distances and accumulate in the axillary buds within a timeframe that correlates with bud release after the loss of the shoot tip. These authors also reported that artificially increasing sucrose levels in plants represses the expression of *BRANCHED1* (*BRC1*), the key transcriptional regulator responsible for maintaining bud dormancy, and results in rapid bud release.^56^ In our previous study, we also observed that many genes associated with sucrose metabolism, such as raffinose synthase family protein, sucrose synthase 4, and plant neutral invertase family protein, displayed altered expression profiles. We believe that sucrose plays important roles in both metabolism and signaling during dormancy release. During the early stage, sucrose may function as a signal to induce dormancy release and may then have metabolic functions.

Gibberellins promote starch degradation by increasing α-amylase activity in seeds. Recently, α-amylase genes were also reported to play important roles in bud dormancy release.^58^ Analyzing the primary reason leading to a high increase in the sucrose level after GA_4_ treatment, whether it be starch degradation, specific *SPS* up-regulation or both, is of interest. In our study, we performed an expression analysis of three genes associated with sucrose metabolism (*INVERTASE*, *SPS*, and *SS*) and found that their expression changed in a manner that was consistent with changes in the sucrose content (Table 1, Figure 4). Our previous study also reported that the relative expression level of *β-amylase 6* decreased more than 2-fold after 10 days of GA_4_ treatment.^10^ Unfortunately, we did not detect any change in *α-amylase* at either the proteomic or transcriptomic level after 5 days of GA_4_ treatment in our previous study. However, we also believe that the large increase in sucrose levels after GA_4_ treatment might be due to both starch degradation and specific *SPS* up-regulation.

Polyols (sugar alcohols) are the reduced forms of aldose and ketose sugars. Polyols are involved in stabilizing macromolecules and scavenging hydroxyl radicals, thereby contributing to the prevention of oxidative damage of enzymes and membranes.^59^ Accumulation of the straight-chain polyols mannitol and sorbitol has also been reported to correlate with stress tolerance in several plant species.^60^ Numerous roles have been attributed to polyols, such as the translocation and storage of photosynthates.^61^ Various studies have reported an increase in polyol content in plants in response to abiotic stresses such as drought, extreme temperature, or salinity. Interest in studying the metabolism of soluble sugar compounds including alditols (myo-inositol, mannitol, sorbitol, dulcitol, and galactinol) is growing. In the present study, we also observed that many sugars and polyols such as threitol, D-arabitol, myo-inositol, and mannitol showed dynamic changes in abundance after GA_4_ and water treatments and that these metabolites showed larger changes in abundance after GA_4_ treatment than after water treatment, indicating that they might play roles in dormancy release following GA_4_ treatment. In our previous study, proteins associated with the metabolism of sugars and polyols, such as alcohol dehydrogenase 1, aldolase superfamily protein, D-3-phosphoglycerate dehydrogenase, myo-inositol-1-phosphate synthase 2, and putative triosephosphate isomerase showed significant changes at the proteomic level after GA_4_ treatment. Many genes associated with the metabolism of sugars and polyols, such as *inositol transporter 4*, *polyol/monosaccharide transporter 5*, *GroES-like zinc-binding alcohol dehydrogenase family protein*, *Ribose 5-phosphate isomerase*, and *type A protein*, also showed significant changes at the transcriptomic level. Those results indicated that polyols might play roles in dormancy release following GA_4_ treatment.

Fatty acids have been reported to play essential roles in plant development and protection. Fatty acids are widely found as chemical energy reserves and major carbon sources in seeds.^62^ However, thus far, no report has examined the relationships between metabolites associated with fatty acids and dormancy release in fruit trees. In our study, we observed that the changes in metabolite abundance during GA_4_ treatment were larger and faster than those during water treatment. However, the effects of metabolites associated with fatty acids on dormancy release in Japanese apricot require further investigation.

In our study, many metabolites associated with galactose, starch, sucrose, glyoxylate, dicarboxylate, fructose, and mannose metabolism, with photosynthetic carbon fixation in nitrogen metabolism, and with carbohydrate digestion and absorption have different abundances after GA_4_ treatment. In particular, the induction of metabolites involved in galactose metabolism, starch and sucrose metabolism, and glyoxylate and dicarboxylate metabolism might contribute the energy needed for dormancy release. In agreement with our results, Maurel *et al*. reported that the transition from dormancy to active bud growth is accompanied by numerous molecular and biochemical changes, including changes in carbohydrate metabolism.^63^ Other studies have also suggested that bud meristems obtain sufficient energy from the underlying tissue to sustain bud growth at the time of dormancy release.^10,33,64,65^

At present, detecting the instantaneous date of bud dormancy release remains difficult. Chilling requirement and budburst are two common indicators analyzed when researchers study dormancy release.^17,29,66,67^ However, budburst is one step in the sequence of dormancy release, and budburst is the indicator that dormancy has been released. In our study, we observed that the percentage of flower buds showing budburst was 60.0% ± 2.5% after 10 days of GA_4_ treatment, but 20.0% ± 1.0% after 10 days of water treatment, indicating that GA_4_ treatment can promote dormancy release. Recently, Vitasse and Basler clearly demonstrated that watered cuttings are better surrogates than are juvenile trees for assessing the potential phenological responses of temperate forests to climate change in warming and photoperiod experiments, and no significant phenological discrepancy was found between cuttings and donor trees when comparing the thermal time to budburst; therefore, shoot detachment might play a slight role in dormancy release.^68^ Overall, we believe that GA_4_ treatment induced bud dormancy release rather than shoot detachment.

## Conclusion

We evaluated the effect of GA_4_ treatment on the metabolism of Japanese apricot and classified metabolites with more than two-fold abundance changes after treatment into six groups: amino acids and their isoforms (8), amino acid derivatives (6), sugars and polyols (12), organic acids (8), fatty acids (3), and others (7). Those metabolites are primarily involved in galactose, glyoxylate, dicarboxylate, starch, and sucrose metabolism may play important roles in GA_4_-induced dormancy release, suggesting that energy metabolism might function at the metabolic level in dormancy release after GA_4_ treatment. Owing to the significant changes in the abundances of metabolites such as sucrose, proline, linoleic acid, and linolenic acid after GA_4_ treatment, these metabolites may function during the dormancy process, and further analyses of the roles of these metabolites may clarify the molecular mechanism of dormancy in Japanese apricot. This research extends our understanding of the dormancy mechanisms in Japanese apricot and provides a theoretical basis for applying GA_4_ to break dormancy.

## Figures and Tables

**Table 1:** Identification of 44 metabolites associated with dormancy release in Japanese apricot treated with GA_4_

Classification of metabolites	Var ID	Peak	Retention time (min)	Fragments (m/z)	Fold changes	
					G5/W0	G10/G5
Amino acids and their isoforms (8)	11	Glycine-TMS	7.63	102	+4.09ab	−3.06b
	24	Norleucine-TMS	11.17	158	+2.57a	−1.98a
	27	Proline-TMS	11.82	142	+27.79a	−4.05a
	36	Serine-TMS	13.38	204	+5.68a	−1.06a
	52	Beta-alanine-TMS	14.95	174	−6.29a	+2.06a
	107	Glutamic acid-TMS	19.53	128	+5.00a	+1.22a
	118	Asparagine-TMS	20.80	116	−1.50a	−68.39a
	203	Tyrosine-TMS	26.37	218	−1.44a	−2.77a
Amino acid derivatives (6)	28	3-aminoisobutyric acid-TMS	12.05	174	+5.07bc	−1.01c
	32	Cycloleucine-TMS	12.76	156	+3.05a	−1.22a
	40	3-cyanoalanine-TMS	13.63	141	−19.06	+1.01
	399	Epicatechin-TMS	41.58	179	−1.00a	−2.56a
	163	Citrulline-TMS	23.99	117	+2.76a	+1.76a
	78	3-hydroxy-L-proline-TMS	17.41	230	−3.25	−1.20
Sugars and polyols (12)	84	Threitol-TMS	17.90	219	−1.94a	−46.67a
	116	Lyxose-TMS	20.68	103	+8.70a	−1.70b
	129	Levoglucosan-TMS	21.61	204	+36.94bc	−1.19bc
	201	D-talose-TMS	26.28	205	−4.26c	+1.05a
	189	Ribose-TMS	25.59	217	+3.36	−1.80
	251	Myo-inositol-TMS	29.59	217	−1.73a	−24.07a
	261	Mannitol-TMS	30.34	205	−2.05a	−1.37a
	371	Lactose-TMS	39.31	204	−1.28a	−44.55a
	389	Cellobiose-TMS	40.78	160	−4.56	−2.98
	394	Maltotriitol-TMS	41.16	204	−46.10	+1.45
	398	Trehalose-TMS	41.56	169	−5.88	+1.57
	449	Sucrose-TMS	46.51	169	+13.86a	−7.50a
Organic acids (8)	38	Pipecolinic acid-TMS	13.47	156	−2.12	+1.49
	146	Saccharic acid-TMS	22.82	217	−2.10c	+1.08ab
	151	Galactonic acid-TMS	23.09	205	−2.87b	+1.35a
	153	Terephthalic acid-TMS	23.20	221	+3.29	−1.25
	164	Citric acid-TMS	24.07	147	−22.28	−1.15
	181	Quinic acid-TMS	25.10	204	−4.66	−1.79
	419	Vanillylmandelic acid-TMS	43.13	299	−3.82a	+3.32a
	421	Chlorogenic acid-TMS	43.31	219	−1.20b	−6.32b
Fatty acids (3)	269	Linoleic acid-TMS	31.11	67	−2.24	−133.09
	270	Oleic acid-TMS	31.19	145	−23.82	+1.40
	272	Linolenic acid-TMS	31.25	79	−22.69b	+23.26a
Others (7)	165	Methyl-beta-D-galactopyranoside-TMS	24.23	204	−2.73a	−1.94a
	192	Glucose-1-phosphate-TMS	25.76	217	−3.34	+1.27
	233	Beta-mannosylglycerate-TMS	28.38	204	−8.35b	−1.05b
	338	1-monopalmitin-TMS	37.33	57	+10.65a	−8.30a
	147	D-(glycerol 1-phosphate)-TMS	22.88	299	−2.66b	−12.62b
	441	Galactinol-TMS	45.43	191	+7.99a	−3.98a
	20	Urea-TMS	10.22	189	+10.52	+1.71

The relative concentration of each metabolite is presented as the mean of data from six biological replicates and was determined using GC–MS. Only those metabolites that showed a more than two-fold change or less than 0.5-fold change that was statistically significant as determined by two-way ANOVA (*P* < 0.05) were considered as significant. W0, G5, and G10 represent GA_4_ treatment after 0, 5, and 10 days, respectively. In the Fold changes column, ‘+’ means an increase in metabolite abundance, and ‘–’ means a decrease in metabolite abundance. Values within rows that are followed by the same letter are not significantly different from each other (Tukey’s HSD test, *P* < 0.05).
