# Supplementary material for: Metabolic changes upon flower bud break in Japanese apricot are enhanced by exogenous GA4
Source: Hortic Res. 2015 Sep 30;2:15046–. doi: 10.1038/hortres.2015.46 (PMC4588617; doi:10.1038/hortres.2015.46)
Supplement: Supplementary Figures S1–S5 and Tables S1–S2 [file hortres201546-s1.pdf]

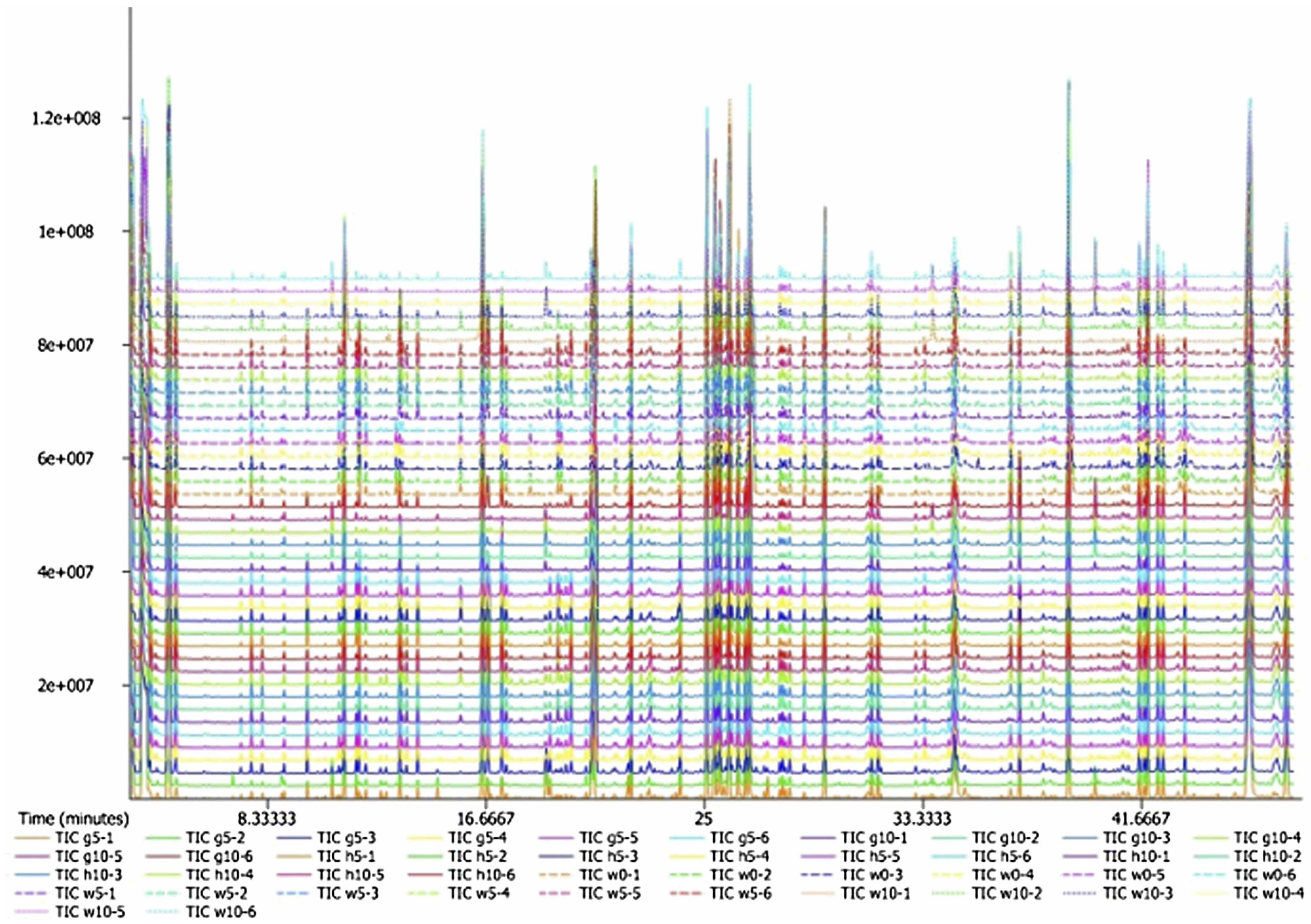


**Supplementary Figure S1** GC–MS TIC for the 42 Japanese apricot flower bud samples.

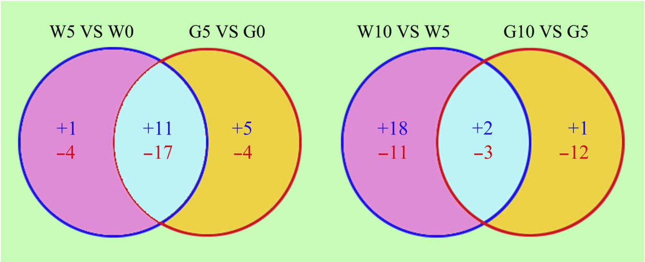


**Supplementary Figure S2** Venn diagrams showing the coordinate changes in metabolite abundance after $GA_4$ and water treatment. Diagrams are based on a set of 55 identified metabolites showing different abundances after 5 or 10 days of $GA_4$ treatment and after 0, 5, or 10 days of water treatment. '+' indicates the metabolites that increased after treatment, and '−' indicates those that decreased after treatment. The number in the box shows the numbers of metabolites with changed abundance after treatment. W0, W5, and W10 denote $GA_4$ treatment after 0, 5, and 10 days, respectively.

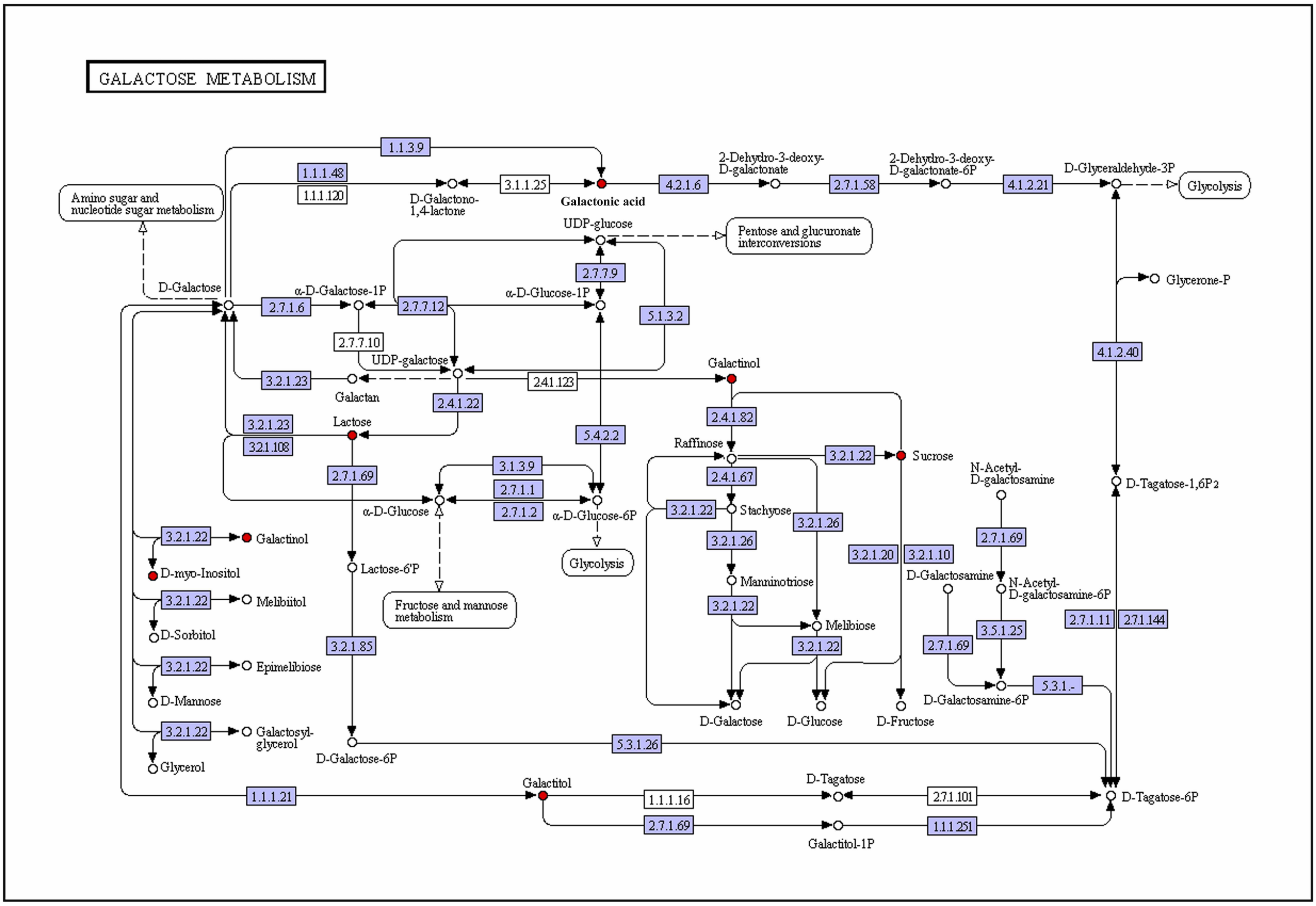


**Supplementary Figure S3** Functional regulation of galactose metabolism is depicted as pathway maps according to the KEGG metabolic database, and the identified metabolites with more than two-fold abundance changes after treatment are represented by red dots. The changes of abundance in the identified metabolites after treatment are shown in Table 1 and Supplementary Table S2.

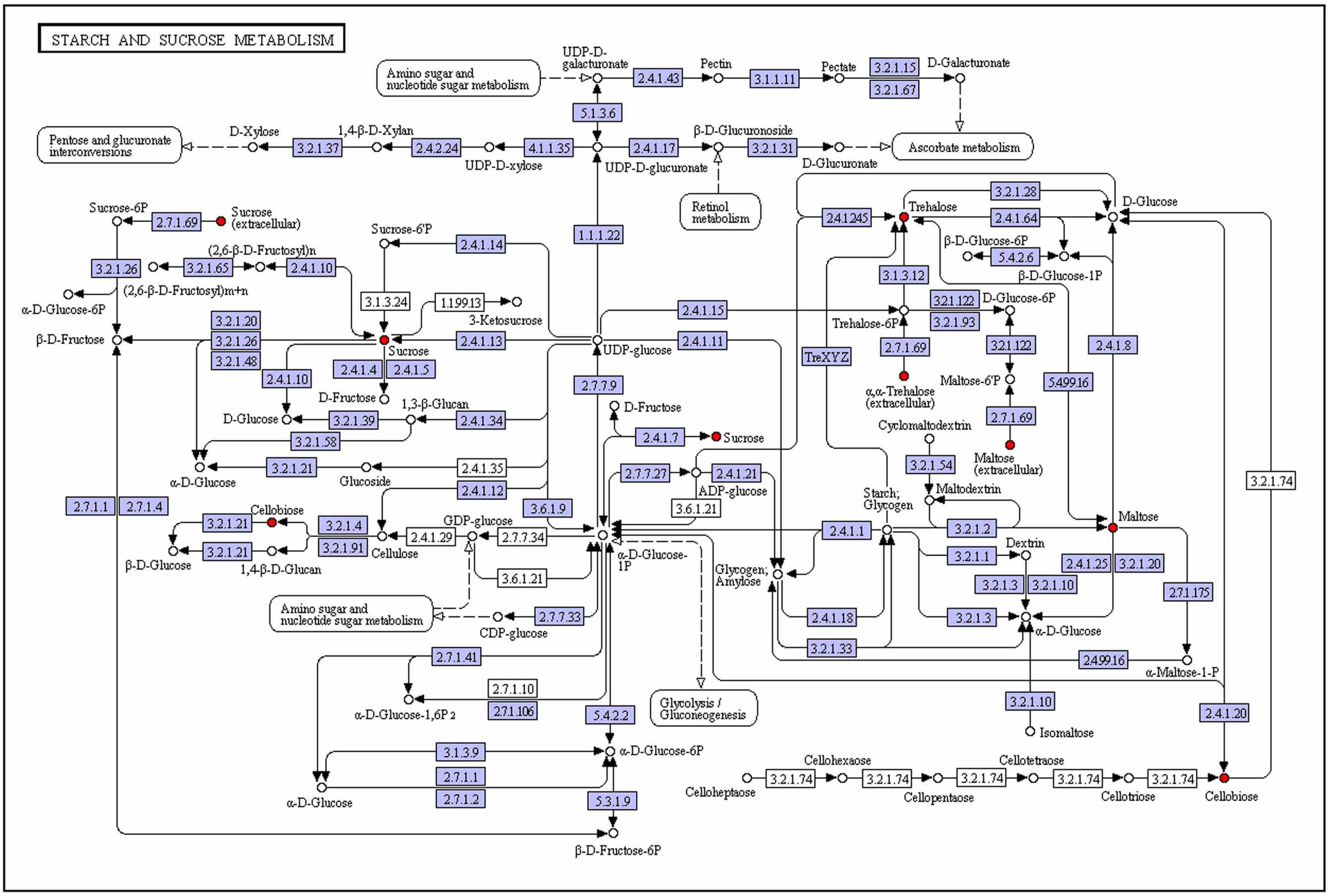


**Supplementary Figure S4** Functional regulation of starch and sucrose metabolism is depicted as pathway maps according to the KEGG metabolic database, and the identified metabolites with more than two-fold abundance changes after treatment are represented by red dots. The changes of abundance in the identified metabolites after treatment are shown in Table 1 and Supplementary Table S2.

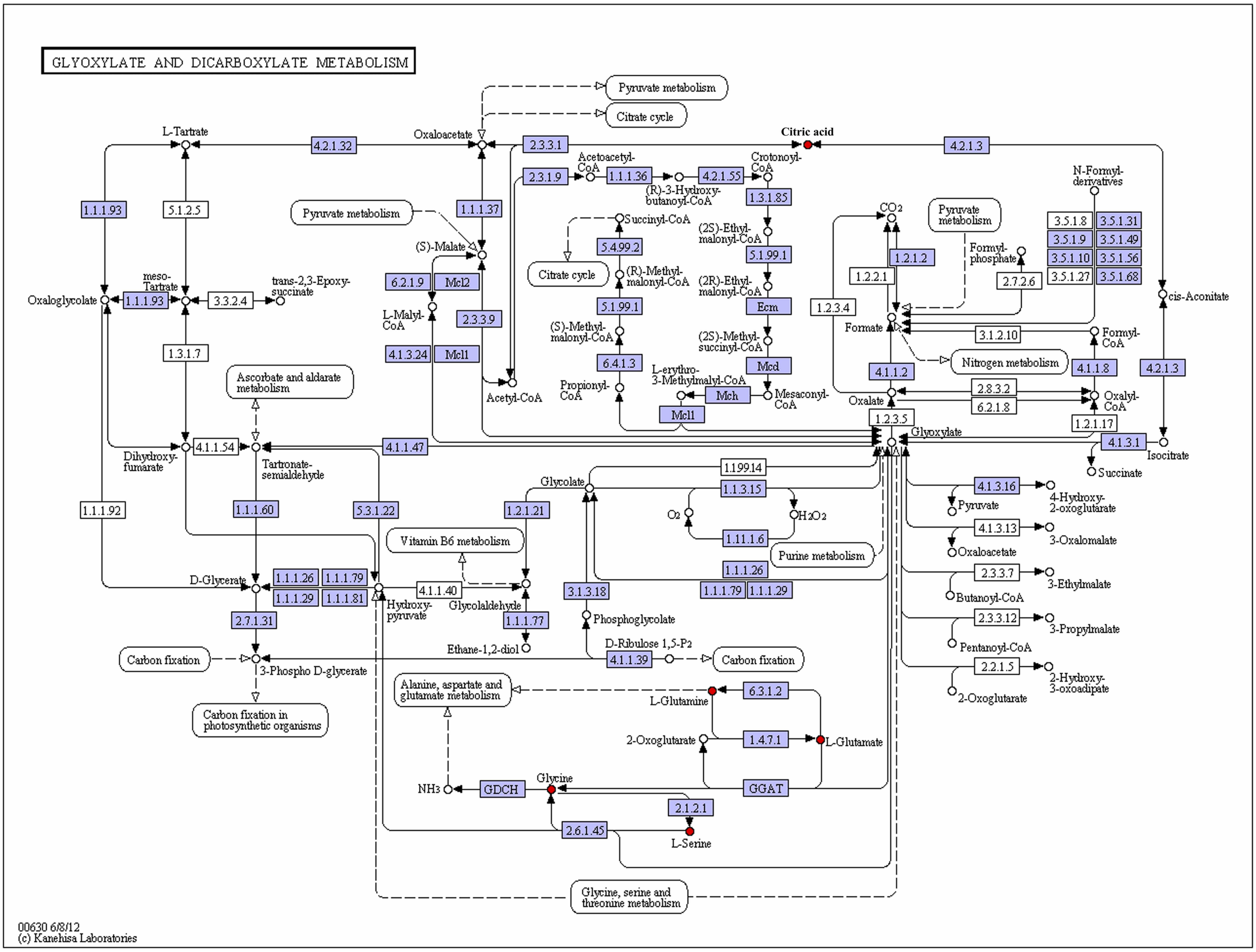


**Supplementary Figure S5** Functional regulation of glyoxylate and dicarboxylate metabolism is depicted as pathway maps according to the KEGG metabolic database, and the identified metabolites with more than two-fold abundance changes after treatment are represented by red dots. The changes of abundance in the identified metabolites after treatment are shown in Table 1 and Supplementary Table S2.

**Table S1** Specific primers used for relative quantitative real-time RT-PCR

| Gene name | Accession | Forward primer Sequence (5'to 3') | Reverse primer Sequence (5' to 3') |
|---|---|---|---|
| cytosolic invertase 2 | Pm018344 | TTCCAGTGCGACGACAACG | TCAATCCTCTTCTCCCAACC |
| sucrose phosphate synthase 1F | Pm004643 | CTATGATACTTGCCCTTGC | TGCTGCCAGACGATAAATG |
| sucrose synthase 4 | Pm027136 | CTGAGGAAGGCAGAGGATTAC | GAGCGAAATAGCCGTGGG |
| α-amylase | PmC022906 | GGGAGCAGTGTGACTATTTT | AAGGTCTCCAAGGTCCGAT |

**Table S2** Identification of 46 metabolites associated with dormancy release in Japanese apricot treated with water. The relative concentration of each metabolite is a mean of data from six biological replicates using GC-MS. Only those metabolites that showed more than two-fold or less than 0.5-fold change and statistically significant in two-way ANOVA ($P < 0.05$) were considered as significant metabolite changes. W0, W5 and W10 represent water treatment after 0 d, 5 d and 10 d, respectively. In the column, '+' means an increase in metabolites abundance and '–' means a decrease in metabolites abundance. Values within rows followed by the same letters are not significantly different (Tukey's HSD test, $p < 0.05$)

| Classification of metabolites | Var ID | Peak | Retention Time (min) | Fragments (m/z) | Fold changes | |
|---|---|---|---|---|---|---|
| | | | | | W5/W0 | W10/W5 |
| Amino acids and their isoforms (9) | 11 | Glycine-TMS | 7.63 | 102 | −2.06b | +13.75a |
| | 24 | norleucine-TMS | 11.17 | 158 | +2.90a | −4.04a |
| | 27 | proline-TMS | 11.82 | 142 | +5.83a | +2.27a |
| | 36 | serine-TMS | 13.38 | 204 | +6.81a | −7.62a |
| | 52 | beta-alanine-TMS | 14.95 | 174 | +1.04a | −2.84a |
| | 76 | aspartic acid-TMS | 17.26 | 218 | +1.17b | −4.83b |
| | 107 | glutamic acid-TMS | 19.53 | 128 | +4.98a | −2.75a |
| | 118 | asparagine-TMS | 20.80 | 116 | −3.01a | +2.07a |
| | 203 | tyrosine-TMS | 26.37 | 218 | +1.12a | −2.06a |
| Amino acid derivatives (6) | 9 | sarcosine-TMS | 7.31 | 116 | +1.06a | −3.78a |
| | 28 | 3-aminoisobutyric acid-TMS | 12.05 | 174 | 174 | −4.89c |
| | 32 | 3-cyanoalanine-TMS | 13.63 | 141 | +2.04a | +3.99a |
| | 40 | 3-cyanoalanine-TMS | 13.63 | 141 | −3.19 | −1.28 |
| | 399 | epicatechin-TMS | 41.58 | 179 | −36.33a | +27.45a |
| | 78 | 3-hydroxy-L-proline-TMS | 17.41 | 230 | −5.64 | +1.24 |
| Sugars and polyols (12) | 116 | lyxose-TMS | 20.68 | 103 | +5.37ab | −1.56b |
| | 129 | levoglucosan-TMS | 21.61 | 204 | −1.31c | +9.16bc |
| | 201 | D-talose-TMS | 26.28 | 205 | −2.68c | −1.37b |
| | 211 | D-arabitol-TMS | 26.82 | 218 | −1.17a | −2.99a |
| | 189 | ribose-TMS | 25.59 | 217 | +4.31 | −1.15 |
| | 251 | myo-inositol-TMS | 29.59 | 217 | −1.01a | −2.85a |
| | 261 | mannitol-TMS | 30.34 | 205 | −2.40a | +2.67a |
| | 371 | lactose-TMS | 39.31 | 204 | −4.31a | −1.22a |
| | 389 | cellobiose-TMS | 40.78 | 160 | −4.45 | +1.13 |
| | 394 | maltotriitol-TMS | 41.16 | 204 | −5.55 | −1.12 |
| | 398 | trehalose-TMS | 41.56 | 169 | −6.99 | +7.36 |
| | 449 | sucrose-TMS | 46.51 | 169 | +1.77a | +17.01a |
| Organic acids (9) | 141 | glucoheptonic acid-TMS | 22.57 | 205 | +1.52b | +9.26a |
| | 151 | galactonic acid-TMS | 23.09 | 205 | −2.39b | −1.45ab |
| | 153 | terephthalic acid-TMS | 23.20 | 221 | +1.62 | +3.75 |
| | 160 | shikimic acid-TMS | 23.75 | 204 | +1.05c | −2.13c |
| | 164 | citric acid-TMS | 24.07 | 147 | −32.65 | +8.12 |
| | 181 | quinic acid-TMS | 25.10 | 204 | −16.46 | +3.09 |
| | 232 | gluconic acid-TMS | 28.24 | 217 | −1.09c | −4.10c |
| | 419 | vanillylmandelic acid-TMS | 43.13 | 299 | −2.21a | +2.55a |
| | 421 | chlorogenic Acid-TMS | 43.31 | 219 | −1.13b | −4.96b |
| Fatty acids (4) | 269 | linoleic acid-TMS | 31.11 | 67 | −4.06 | −1.16 |
| | 229 | palmitic acid-TMS | 28.08 | 117 | +2.86a | −3.42a |
| | 270 | oleic acid-TMS | 31.19 | 145 | −31.50 | +5.76 |
| | 272 | linolenic acid-TMS | 31.25 | 79 | −2.67b | +2.61ab |
| Others (6) | 192 | glucose-1-phosphate-TMS | 25.76 | 217 | −2.75 | +7.82 |
| | 233 | beta-Mannosylglycerate-TMS | 28.38 | 204 | −25.41b | +18.04a |
| | 338 | 1-Monopalmitin-TMS | 37.33 | 57 | +30.97a | +1.59a |
| | 147 | D-(glycerol 1-phosphate) -TMS | 22.88 | 299 | −4.85b | +4.03a |
| | 441 | galactinol-TMS | 45.43 | 191 | +2.02a | +8.26a |
| | 20 | urea-TMS | 10.22 | 189 | +12.51 | +1.34 |
